# Comparative systemic pathogenicity and host tropism of ancestral SARS-CoV-2, Delta, and Omicron EG.5 variants in K18-hACE2 mice

**DOI:** 10.3389/fimmu.2026.1907444

**Published:** 2026-08-11

**Authors:** Xiaofang Peng, Qi Zhang, PingPing Zhou, Li Tang, Chumin Liang, Huifang Lin, Zhe Liu, Baisheng Li, Jiufeng Sun, Runyu Yuan

**Affiliations:** 1 Guangdong Workstation for Emerging Infectious Disease Control and Prevention, Guangdong Provincial Key Laboratory of Pathogen Detection for Emerging Infectious Disease Response, Guangdong Provincial Center for Disease Control and Prevention, Guangzhou, China; 2 Key Laboratory of Zoonosis Prevention and Control of Guangdong, College of Veterinary Medicine, South China Agricultural University, Guangzhou, China; 3 Guangdong Provincial Institute of Public Health, Guangzhou, China

**Keywords:** K18-hACE 2 transgenic mice, neurotropism, Omicron, pathogenicity, SARS-CoV-2, viral variants

## Abstract

**Introduction:**

SARS-CoV-2 undergoes persistent genomic evolution, yet prevalent circulating variants tend to evolve toward reduced pathogenicity. However, direct comparative pathogenesis data involving contemporary, immune-evasive strains—such as the Omicron EG.5 subvariant (a descendant of the globally dominant XBB lineage)—relative to ancestral strains remain scarce.

**Methods:**

To clarify the evolutionary trajectory of viral virulence, we conducted a systematic, head-to-head comparison of the in vivo pathogenicity of three pivotal strains: the ancestral Wild-type (Wuhan lineage), Delta, and the currently circulating Omicron EG.5. We utilized the highly susceptible K18-hACE2 transgenic mouse model to assess disease progression, viral burden, and histopathology.

**Results:**

Despite uniformly progressing to lethality in this model, the strains exhibited a distinct gradient in the kinetics and severity of disease. The Wild-type strain demonstrated the most rapid disease progression, highest viral loads across multiple organs (notably liver, brain, lung, and intestine), and most severe histopathological damage. The Delta variant showed an intermediate phenotype. Strikingly, the Omicron EG.5 variant displayed significantly attenuated pathogenicity, characterized by markedly delayed mortality, milder clinical presentation, and reduced tissue viral burden, yet it retained a capacity for multi-organ infection.

**Discussion:**

Our study provides direct experimental evidence for a pronounced attenuation in pathogenicity along the evolutionary trajectory from Wild-type through Delta to Omicron EG.5. These findings offer crucial insights for risk assessment of emerging variants and underscore the need for ongoing surveillance.

## Introduction

The persistent evolution of severe acute respiratory syndrome coronavirus 2 (SARS-CoV-2) presents an unprecedented challenge to global public health, driven by the continual emergence of variants with altered transmissibility, immune evasion, and pathogenicity (1, 2). Since its initial identification, the virus has diversified from the ancestral Wild-type (WT, Wuhan-Hu-1) strain into multiple variants of concern, variants of interest, and variants under monitoring, including Alpha (B.1.1.7), Beta (B.1.351), Gamma (P.1), Delta (B.1.617.2), and Omicron sublineages (3–6). Notably, the Delta variant was associated with significantly enhanced virulence, leading to increased risks of hospitalization and severe disease (7). A fundamental shift in the pandemic landscape occurred with the emergence of the Omicron variant (B.1.1.529) and its numerous sublineages (8, 9). Characterized by an extensive number of spike protein mutations, Omicron variants exhibit extraordinary transmissibility and a profound ability to escape pre-existing immunity (10, 11). Concurrently, extensive epidemiological evidence indicates a marked attenuation in their intrinsic pathogenicity compared to prior variants, with reported reductions of 59-80% in hospitalization risk and approximately 70% in severe disease risk relative to Delta (12–14). However, while clinical data suggest reduced severity, the extent to which this attenuation extends to tissue tropism-particularly in the central nervous system and gastrointestinal tract-remains poorly defined for contemporary Omicron subvariants.

While population-level data clearly suggest reduced clinical severity, a precise, mechanistic understanding of the attenuated pathogenicity of Omicron sublineages-particularly contemporary strains like Omicron EG.5 (a descendant of XBB)-remains less defined. Controlled, comparative studies in susceptible animal models are crucial to disentangle the intrinsic viral factors from population-level immunity and to systematically evaluate tissue tropism, replication kinetics, and pathogenicity. The K18-hACE2 transgenic mouse model, which expresses the human angiotensin-converting enzyme 2 (hACE2) receptor, is a well-established and sensitive system for studying SARS-CoV-2 pathogenesis, faithfully recapitulating key features of severe human disease (15–18). This model provides a standardized platform for head-to-head comparisons of viral virulence under identical experimental conditions.

While accumulating clinical data suggest attenuated severity for Omicron sublineages, controlled comparative studies evaluating the contemporaneous Omicron EG.5 variant remain limited. Specifically, the retention of neurotropism and multi-organ tropism in Omicron EG.5 compared to earlier, more virulent strains (Wild-type and Delta) require systematic evaluation in a standardized model. To address this, we conducted a comparative analysis of the ancestral Wild-type, Delta, and Omicron EG.5 variants using K18-hACE2 transgenic mice. This study provides a comprehensive assessment of virulence gradients, viral replication kinetics, and histopathological damage across multiple organ systems, offering critical insights into the evolving pathogenic profile of SARS-CoV-2.

## Methods

### Viruses

Three clinically isolated SARS-CoV-2 strains representing distinct genotypes were utilized in this study: the ancestral Wild-type (Wuhan lineage, preservation no. GDPCC-nCOV1), the Delta variant (preservation no. GDPCC 2.00118), and the Omicron EG.5 subvariant (preservation no. GDPCC 2.02228). All viral strains were originally isolated from patient specimens (nasopharyngeal swabs or sputum) in Guangdong Province, China, between 2020 and 2023, and were provided by the Culture Collection Center of Virus and Bacteria Strain at the Guangdong Provincial Center for Disease Control and Prevention.

### Sequence analysis

Multiple sequence alignment of representative viruses was performed together with alignment of sequences downloaded from GenBank. Full-length gene sequences were used for phylogenetic analyses. The generated model was used in all subsequent analyses. Neighbor-joining and maximum-likelihood trees were constructed to use MEGA program (version 12) with 1,000 bootstraps (19). All of the branches supported by >50% bootstrap values were considered to be in the same group in the trees.

### Animals, grouping, and viral infection

Six- to eight-week-old specific pathogen-free (SPF) K18-hACE2 transgenic mice (GemPharmatech Co., Ltd., China) were used in this study. GemPharmatech independently developed an ACE2 humanized mouse model using gene editing technology to simulate the clinical manifestations of human infection with SARS-CoV-2. Based on the C57BL/6JGpt background strain, ACE2 was humanized. Specifically, the human cytokeratin 18 (Cytokeratin 18, K18) promoter was utilized to drive the overexpression of hACE2 at the H11 safe locus, thereby establishing a model to simulate severe human COVID-19 phenotypes. Mice were randomly assigned to four experimental groups (n=9 per infected group, n=3 for the Mock control): the Wild-type strain group, the Delta variant group, the Omicron EG.5 variant group, and a Mock-infected control group. Three mice per infected group were dedicated to daily monitoring of body weight dynamics and survival outcomes. The remaining six animals in each group were humanely euthanized at 3 dpi for terminal tissue harvest, followed by viral RNA load quantification and histopathological assessments via H&E and IHC staining. All mice were housed under standard SPF conditions. Following anesthesia with isoflurane, mice were inoculated intranasally with 30 μL of virus suspension containing 10^6^ 50% cell culture infectious dose (CCID_50_) of the respective SARS-CoV-2 strain. Mock-infected controls received an equal volume of sterile PBS. Clinical signs and body weight were monitored and recorded daily. All procedures involving live SARS-CoV-2 were performed in an Animal Biosafety Level 3 (ABSL-3) laboratory at the Guangdong Provincial Center for Disease Control and Prevention. The animal study protocol was reviewed and approved by the Ethical Review Subcommittee for Animal Experiments and Human Trials of Health-Related Products, Guangdong Provincial Center for Disease Control and Prevention (Approval No. 2023020).

### Histopathological analysis

Tissue specimens encompassing the lungs, heart, liver, spleen, kidneys, brain, small intestine, large intestine, and pancreas were fixed in 4% paraformaldehyde, paraffin-embedded, and cut into 4–5 μm serial sections. Following routine histological processing, sections were dewaxed, rehydrated, stained with hematoxylin and eosin (H&E), dehydrated, cleared, and mounted with neutral balsam for light microscopic observation. All histological evaluations were completed in a blinded manner by a board-certified veterinary pathologist. A modified 0–4 semi-quantitative scoring system was adopted to grade general tissue lesion severity, as previously described (20). The grading standards were defined as follows: 0, intact normal tissue architecture; 1 (minimal), trivial focal lesions marginally deviating from physiological status; 2 (mild), distinct localized lesions without apparent organ functional impairment; 3 (moderate), marked pathological lesions accompanied by visible tissue damage and early organ dysfunction; 4 (severe), extensive coalescing lesions causing severe tissue structural collapse and irreversible functional loss.

Three core pathological parameters were assessed for scoring: (1) inflammatory cell infiltration, (2) tissue edema and/or vascular congestion, and (3) parenchymal necrosis or cellular degeneration. At least five random high-power fields (×400 magnification) were captured and analyzed per section. Within each field, scores of the three individual parameters were averaged to obtain a representative value for each index. The averaged values of the three parameters were summed to yield a composite pathological score ranging from 0 to 9, where higher scores indicated aggravated tissue injury. Pulmonary lesions were further quantified via two separate semi-quantitative scoring systems specific to acute lung injury (ALI) and diffuse alveolar damage (DAD), both assessed blindly on the same lung sections. First, a weighted ALI scoring algorithm was utilized. Three randomly selected microscopic fields per lung section were scored against five histological indicators: (A) Alveolar neutrophil infiltration: 0 = absent; 1 = 1–5 neutrophils per field; 2 = >5 neutrophils per field. (B) Interstitial septal neutrophil infiltration: 0 = absent; 1 = 1–5 neutrophils per field; 2 = >5 neutrophils per field. (C) Hyaline membrane formation: 0 = absent; 1 = single hyaline membrane; 2 = multiple hyaline membranes. (D) Intra-alveolar proteinaceous debris: 0 = absent; 1 = single focal lesion; 2 = multiple focal lesions. (E) Alveolar septal thickening: 0 = thickness < 2-fold of mock control tissue; 1 = 2–4-fold thickening; 2 = >4-fold thickening. A composite field-level lung injury score was calculated with the weighted formula: Field score = [(20 × A) + (14 × B) + (7 × C) + (7 × D) + (2 × E)]/100. This weighted scale quantitatively characterizes histological hallmarks of DAD. Second, a modified 4-tier American Thoracic Society (ATS) grading scale was applied to independently evaluate DAD severity: 1 = No epithelial shedding or parenchymal necrosis. 2 = Sparse isolated foci of epithelial shedding and necrosis (1–2 lesions per field). 3 = Multifocal epithelial injury (≥3 foci) with sporadic alveolar septal hyalinization. 4 = Diffuse epithelial injury involving >75% of the field, accompanied by abundant or prominent hyaline membranes. For each mouse, scores from the three random fields measured by the DAD scale were averaged to generate a unified final DAD severity score representative of the whole lung. This ATS-based scoring framework enables quantitative assessment of murine ALI phenotypes and facilitates translational comparison with pathological features observed in human COVID-19 cases, consistent with prior published protocols (21).

### Immunohistochemical staining and analysis

For immunohistochemistry, tissue samples were fixed in 4% paraformaldehyde, paraffin-embedded, and sectioned (4–5 μm). Following deparaffinization and rehydration, antigen retrieval was performed by heating the sections in citrate buffer (pH 6.0) using a microwave. Endogenous peroxidase activity was quenched by incubation with 3% hydrogen peroxide (H_2_O_2_) for 10 minutes. Sections were then blocked with 5% normal goat serum for 1 hour at room temperature. Subsequently, sections were probed overnight at 4°C with a rabbit anti-SARS-CoV-nucleoprotein polyclonal antibody (Novus Biologicals, Littleton, CO, USA; Cat# NB100-56576) at 1:1000 dilution in blocking buffer. Following three washes with phosphate-buffered saline (PBS), sections were incubated with an HRP-conjugated goat anti-rabbit IgG secondary antibody (Abcam, Cambridge, UK; Cat# ab205718) at a 1:2000 dilution for 30 min at room temperature. Immunoreactivity was visualized using a 3,3′-diaminobenzidine (DAB) substrate kit, with the reaction duration monitored microscopically to prevent overdevelopment. Nuclei were counterstained with hematoxylin. Finally, the sections were dehydrated through graded ethanol series, cleared in xylene, and mounted with neutral balsam. Stained sections were examined and imaged using a light microscope. The expression level of SARS-CoV-2 antigen was quantified by measuring the mean optical density of positive staining in five randomly selected fields per section using image analysis software.

### Viral RNA extraction and quantification

Tissue samples (approximately 50 mg) were collected aseptically at 3 days post-infection. Each sample was homogenized in 1 mL of phosphate-buffered saline (PBS) using a sterile tissue grinder. The homogenate was centrifuged at 12,000 × g for 10 minutes at 4°C, and the resulting supernatant was collected for RNA extraction. Total RNA was extracted from 200 µL of the supernatant using a commercial automated nucleic acid extraction system (Xi’an Tianlong Technology Co., Ltd., China) according to the manufacturer’s protocol. The concentration and purity of the extracted RNA were assessed using a spectrophotometer.

The viral RNA load was quantified by one-step reverse transcription droplet digital PCR (RT-ddPCR). Briefly, reactions were performed using the One-Step RT-ddPCR Advanced Kit for Probes (Bio-Rad Laboratories, Inc. America) on a QX200 Droplet Digital PCR system (Bio-Rad Laboratories, Inc. America), following the manufacturer’s instructions. Droplets were subsequently read on a QX200 Droplet Reader, and the absolute copy number of viral RNA per 20 µL reaction was analyzed using QuantaSoft Analysis Pro software (Bio-Rad Laboratories, Inc. America).

### Statistical analysis

All statistical analyses were conducted with GraphPad Prism 10.5.0. Quantitative data are expressed as mean ± standard error of the mean (SEM). Survival curves were compared via the log-rank (Mantel-Cox) test. Longitudinal body weight fluctuations and tissue viral loads across experimental groups were assessed by one-way or two-way analysis of variance (ANOVA), with Tukey’s or Dunnett’s *post-hoc* tests applied for pairwise comparisons. Since histopathological scoring data violated normality assumptions, intergroup differences were determined using the Kruskal–Wallis test coupled with Dunn’s multiple comparison test. Statistical significance was defined as p < 0.05. Significance markers are annotated as: *p < 0.05, **p < 0.01, ***p < 0.001, ****p < 0.0001; ns, non-significant.

## Results

### Phylogenetic and molecular characterization of distinct SARS-CoV-2 variants

**Figure 1:**
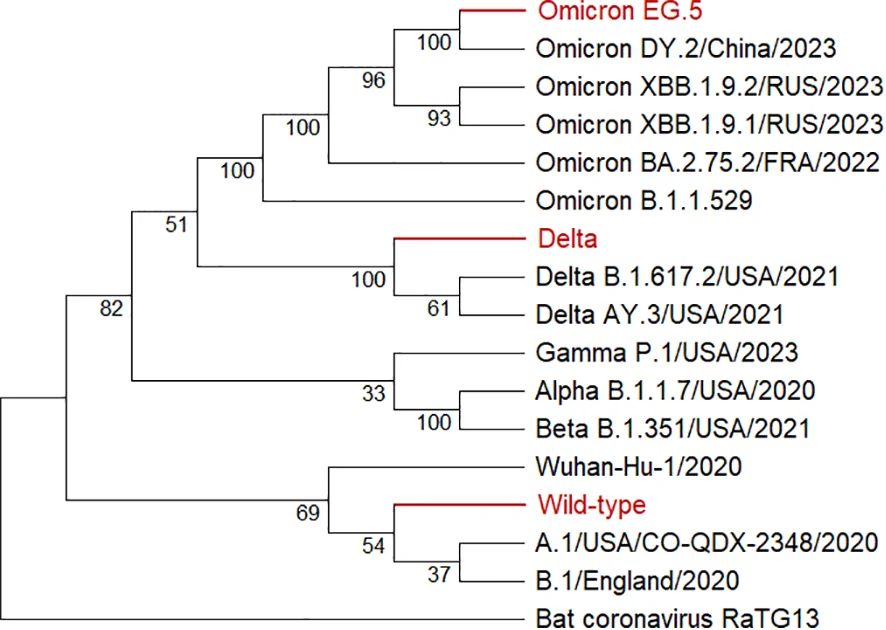
Phylogenetic analysis of distinct SARS-CoV-2 variants. Viruses highlighted with the red were characterized in this study. The tree was constructed using the neighbor-joining method of MEGA 12, with 1,000 bootstrap trials to assign confidence to the groupings.

A phylogenetic tree was constructed to delineate the evolutionary relationships among the studied SARS-CoV-2 strains (**Figure 1**). Using the neighbor-joining method in MEGA 12 software, the analysis confirmed that the Wild-type strain clusters within the ancestral Wuhan-Hu-1 lineage. The Delta variant (B.1.617.2) is phylogenetically closer to earlier variants of concern (VOCs) such as Alpha and Beta. In contrast, the Omicron EG.5 strain forms a distinct cluster within the XBB.1.9.2 sub-lineage, demonstrating its origin from the ongoing diversification of the Omicron lineage.

**Figure 2:**
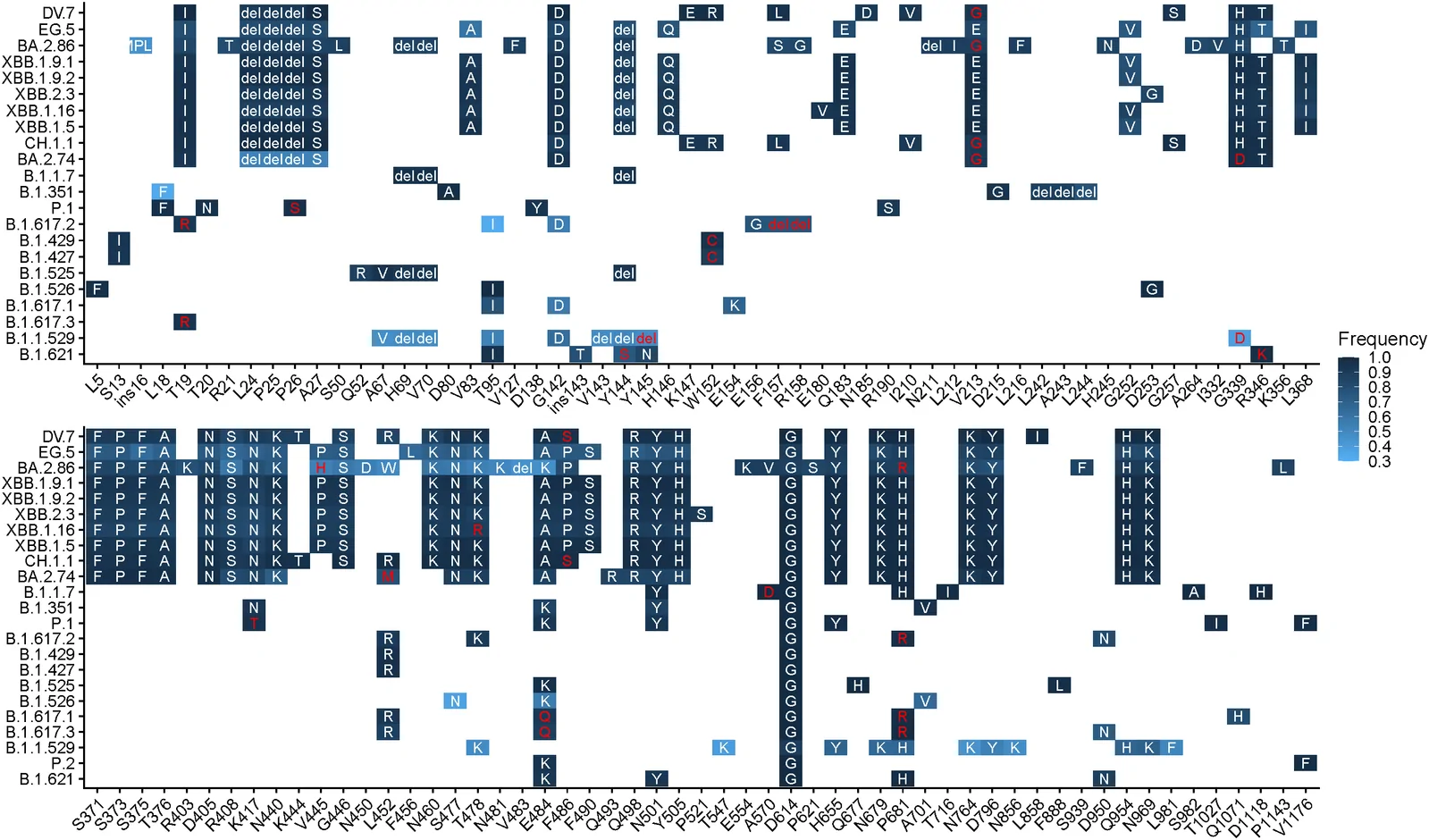
Analysis of spike protein mutation sites and their frequencies in SARS-CoV-2 variants circulating before 2023. Summary of SARS-CoV-2 spike protein mutations across pre-2023 circulating lineages, with all viral sequences retrieved from the Global Initiative on Sharing All Influenza Data hCoV-19 database. Only mutations with >10% population frequency relative to the ancestral wild-type strain are included. Color denotes the prevalence of each mutation per lineage.

Key spike protein mutations defining each variant’s phenotype were identified (**Figure 2**). The ancestral Wild-type strain serves as the baseline for spike-ACE2 receptor binding. The Delta variant harbors characteristic mutations, including L452R, T478K, and P681R, which are collectively associated with enhanced transmissibility, fusogenicity, and pathogenicity, contributing to its global dominance in 2021 (22, 23). The Omicron EG.5 subvariant, descended from BA.2, inherits mutations such as L452R and F486V and has acquired additional substitutions, including F456L and Q52H. This constellation of mutations is known to optimize spike protein conformation, significantly boosting immune evasion and facilitating the global prevalence of this variant (24).

### Graded acute virulence of Wild-type, Delta and Omicron EG.5 in K18-hACE2 mice

**Figure 3:**
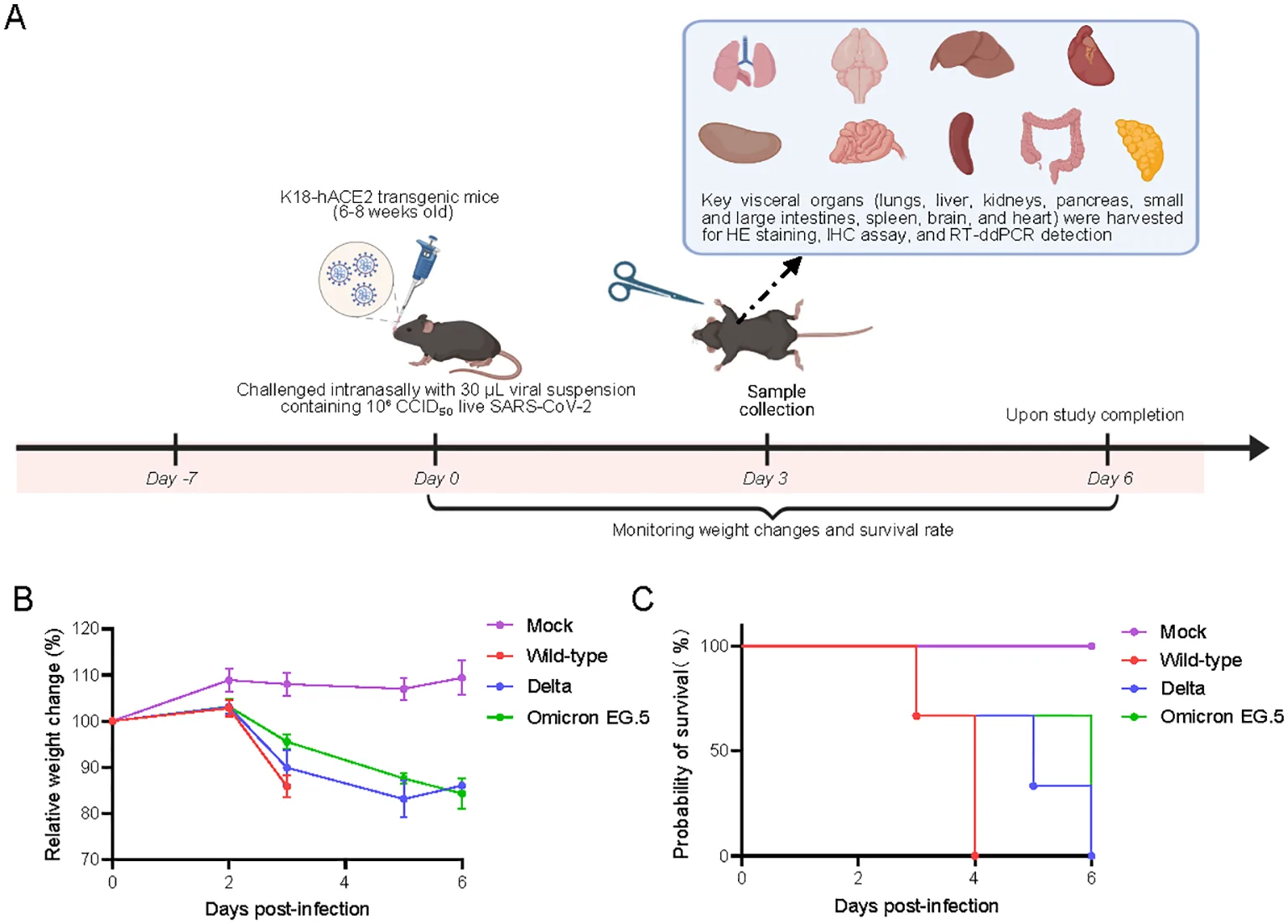
Pathogenicity assessment of distinct SARS-CoV-2 variants in K18-hACE2 transgenic mice. **(A)** Schematic of the experimental design and timeline. K18-hACE2 transgenic mice, backcrossed to the C57BL/6J background, were utilized in this study. **(B, C)** Relative body weight changes **(B)** and survival curves **(C)** of K18-hACE2 transgenic mice following intranasal infection with the indicated SARS-CoV-2 variants (Wild-type, Delta, or Omicron EG.5) or mock treatment. Statistical comparison of survival curves was performed using the log-rank (Mantel-Cox) test.

To assess the pathogenicity of distinct SARS-CoV-2 variants, we intranasally inoculated K18-hACE2 transgenic mice with the Wild-type strain, Delta variant, or Omicron EG.5 variant, and monitored body weight and survival daily (**Figure 3A**). All SARS-CoV-2 strains caused lethal infection in K18-hACE2 mice; however, a striking gradient was observed in the tempo of disease progression and the severity of clinical manifestations among the groups (**Figure 3B**). Mice challenged with the Wild-type strain began losing weight by 3 days post-infection (dpi), exhibiting severe clinical signs including tremor, hunched posture, heightened startle response, and lethargy. Necropsy revealed characteristic cardiopulmonary atrophy, pulmonary congestion, and intestinal bloating. All mice in the Wild-type group succumbed to infection by day 4, resulting in 100% mortality. The maximum body weight loss prior to death ranged from 11.4% to 25% (**Figure 3B**). Infection with the Delta variant also led to rapid weight loss beginning at 3 dpi, accompanied by tremor, hunched posture, and reduced activity. Although the disease course was slightly prolonged compared with the Wild-type group, all Delta-infected mice died by day 6, also yielding 100% mortality. This group exhibited the most pronounced weight loss, with a reduction ranging from 14.1% to 19.7% (**Figure 3B**). In contrast, mice infected with the Omicron EG.5 variant showed a markedly attenuated clinical phenotype. While weight loss commenced at 3 dpi, no obvious severe clinical symptoms were observed throughout the course of infection. Despite this milder presentation, all mice in the Omicron EG.5 group also reached the experimental endpoint by day 6 (100% mortality), with body weight loss ranging from 5.5% to 18%. As expected, mock-infected control mice showed a steady increase in body weight and experienced no mortality (**Figures 3B, C**).

Collectively, these data demonstrate a clear gradient in acute pathogenicity. The Wild-type strain caused the most rapid and severe disease, followed by the Delta variant, which induced the greatest magnitude of weight loss. Notably, despite reaching the same lethal endpoint, the Omicron EG.5 variant presented with significantly delayed mortality and attenuated clinical and pathological severity, defining its reduced virulence in this model.

### Progressive restriction of tissue tropism and alleviated pathological damage alongside SARS-CoV-2 evolution

**Figure 4:**
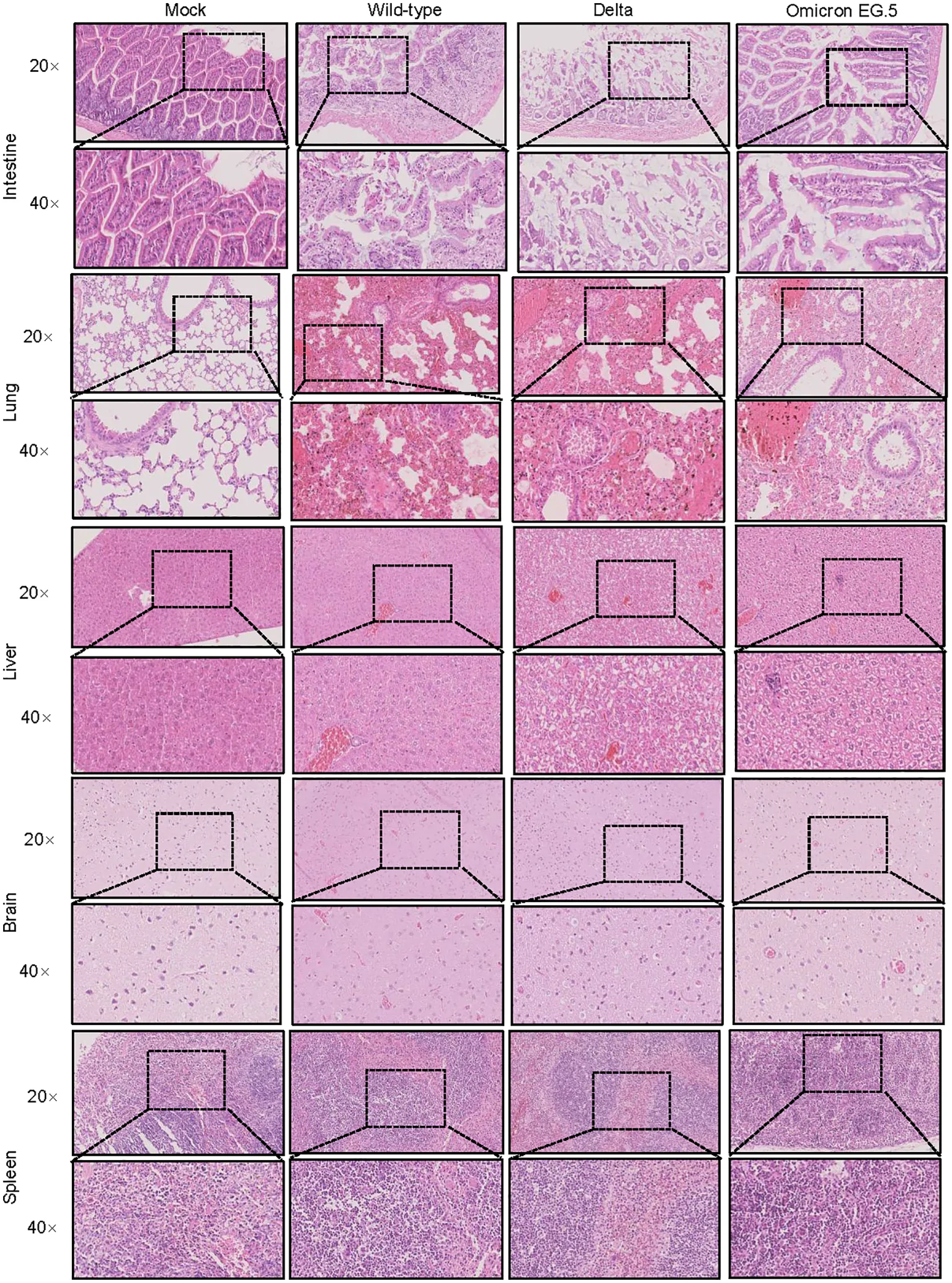
Histopathological characteristics and injury severity of multiple tissues in mice infected with different SARS-CoV-2 variants. Representative hematoxylin and eosin (H&E) staining of intestine, lung, liver, brain, and spleen tissues from mice inoculated with the wild-type strain, Delta variant, Omicron EG.5 variant, or mock-treated control.

**Figure 5:**
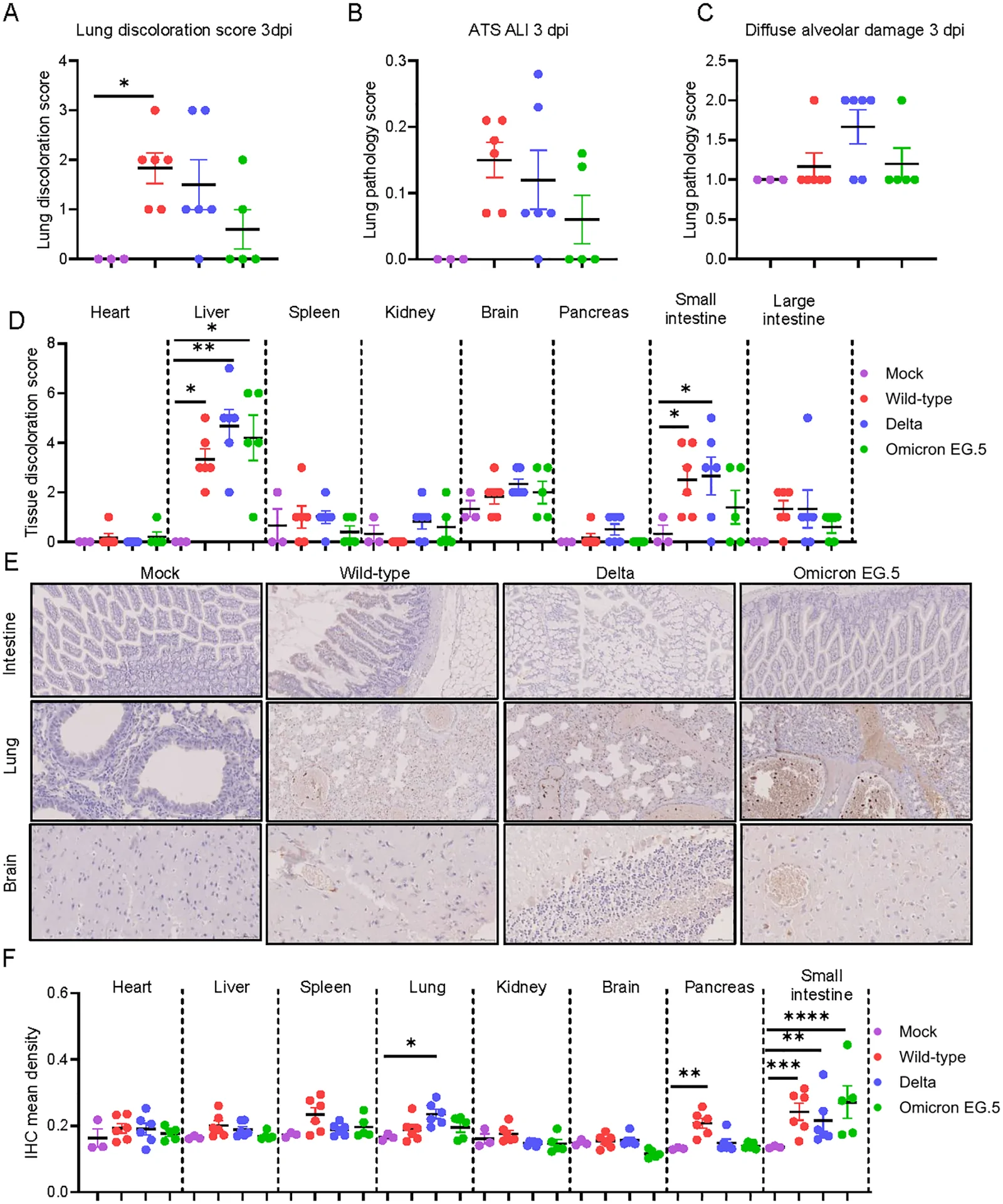
Histopathological characteristics and injury severity of multiple tissues in mice infected with different SARS-CoV-2 variants. **(A)** Macroscopic lung discoloration scores of mice at 3 dpi after challenge with wild-type SARS-CoV-2, Delta or Omicron EG.5 variants, or sterile PBS mock control. **(B)** Lung pathology quantified by ATS acute lung injury (ALI) scores. **(C)** Lung pathology quantified by diffuse alveolar damage (DAD) scores. **(D)** Macroscopic discoloration scores of extrapulmonary tissues. **(E)** Representative IHC staining of intestinal, lung and brain tissues from mice inoculated with wild-type SARS-CoV-2, Delta or Omicron EG.5 variants, or mock control. **(F)** Mean IHC staining density across 8 major organs. Data are shown as mean ± SEM with individual data points; n = 3 (mock), n = 6 (wild-type, Delta), n = 5 (Omicron EG.5, one sample excluded due to processing damage). P values were calculated by one-way ANOVA with Tukey’s *post-hoc* test for panel **(A)**, and by two-way ANOVA with Dunnett’s multiple comparisons test for **(D, F)** *P < 0.05, **P < 0.01, ***P < 0.001, ****P < 0.0001 vs. mock group.

To determine whether the continuous evolution of the virus is associated with a gradual attenuation of tissue tropism and *in vivo* pathogenicity, we performed histopathological examination of multiple organs. Histopathological assessment revealed a clear gradient in the severity and pattern of multi organ injury induced by SARS-CoV-2 infection, correlating with the viral strain (**Figure 4**). The lungs and intestinal tracts were the primary and most severely affected sites. In the lungs, all infected groups exhibited hemorrhage and congestion. We further quantified macroscopic lung discoloration and microscopic lung injury using two independent histological scoring systems, namely the acute lung injury (ALI) and diffuse alveolar damage (DAD) scales, in K18-hACE2 transgenic mice. Markedly alleviated macroscopic lung pathological lesions, accompanied by lower ALI and DAD scores, were observed in mice infected with the Delta or Omicron EG.5 variant relative to animals challenged with the ancestral wild-type SARS-CoV-2 strain (**Figures 5A–C**). In addition, infections with the Delta and Omicron EG.5 variants caused the most pronounced structural damage, including obscured alveolar walls and the presence of necrotic debris. The ancestral Wild-type strain also induced pulmonary hemorrhage and congestion, with occasional granulocyte infiltration. A striking virulence gradient was evident in the intestines. The ancestral Wild-type infection provoked the most severe pathology, characterized by extensive shedding of the mucosal epithelium accompanied by lymphocytic infiltration (**Figures 4**, **5D**). The Delta variant caused severe architectural disruption, with widespread epithelial loss and indistinct glandular structures. In contrast, the Omicron EG.5 variant induced only mild, superficial epithelial shedding. Beyond these primary target organs, systemic involvement with distinct features was observed. The liver displayed hepatocyte edema and moderate vascular congestion in all infected groups, with no marked difference in severity. Mild neuronal edema was consistently present in the brain across groups. A unique pathological finding was necrosis of the splenic white pulp, which occurred exclusively in the ancestral Wild-type infected mice. Other organs, including the heart (mild congestion), kidney (mild tubular epithelial swelling), and pancreas (no significant lesions), showed minimal or non-specific changes that did not differ substantially between infection groups (**Figure 5D**).

IHC analysis quantified the spatial distribution and relative abundance of viral antigen in infected tissues, revealing significant differences in tissue tropism and infectivity among the SARS-CoV-2 strains (**Figure 5E**). The ancestral Wild-type strain exhibited the most extensive and intense tissue distribution. Viral antigen expression, quantified by mean optical density (MOD), was significantly elevated across multiple organs compared with mock infected controls. Notably, the most pronounced signals were detected in the intestine, where MOD values were significantly higher than in controls (P < 0.01), indicating robust replication or substantial viral load in gastrointestinal and associated tissues. The Wild-type strain also showed relatively high MOD values in all other examined organs. Both the Delta and Omicron EG.5 variants demonstrated evidence of more restricted viral dissemination, with significant viral antigen expression primarily confined to the lung and intestinal tissues. Significant viral antigen expression was consistently observed in lung and intestinal tissues, confirming the lung as a primary and conserved target organ and underscoring the retained gastrointestinal tropism of these variants. However, the overall intensity of IHC staining for these variants, particularly for Omicron EG.5, was generally lower than that induced by the ancestral Wild-type strain in comparable tissues (**Figure 5F**).

In summary, the Wild-type strain was associated with the most severe and multifocal pathology, notably in the lungs and intestines, with unique splenic involvement. The Delta variant induced targeted, severe damage in the respiratory and digestive tracts. The Omicron EG.5 variant provoked a qualitatively similar but quantitatively attenuated pathological response, particularly within the intestine, consistent with its reduced clinical virulence. IHC analysis delineated a clear hierarchy in viral tissue distribution and antigen load: Wild-type > Delta > Omicron EG.5. The ancestral Wild-type strain possessed the broadest tissue tropism and highest antigen expression, while the Delta and Omicron EG.5 variants showed a more focal pattern, primarily in the respiratory and gastrointestinal tracts, with attenuated overall infectivity as measured by antigen burden.

### Viral loads sequentially decrease from ancestral strain to Delta and Omicron EG.5 across multiple organs

**Figure 6:**
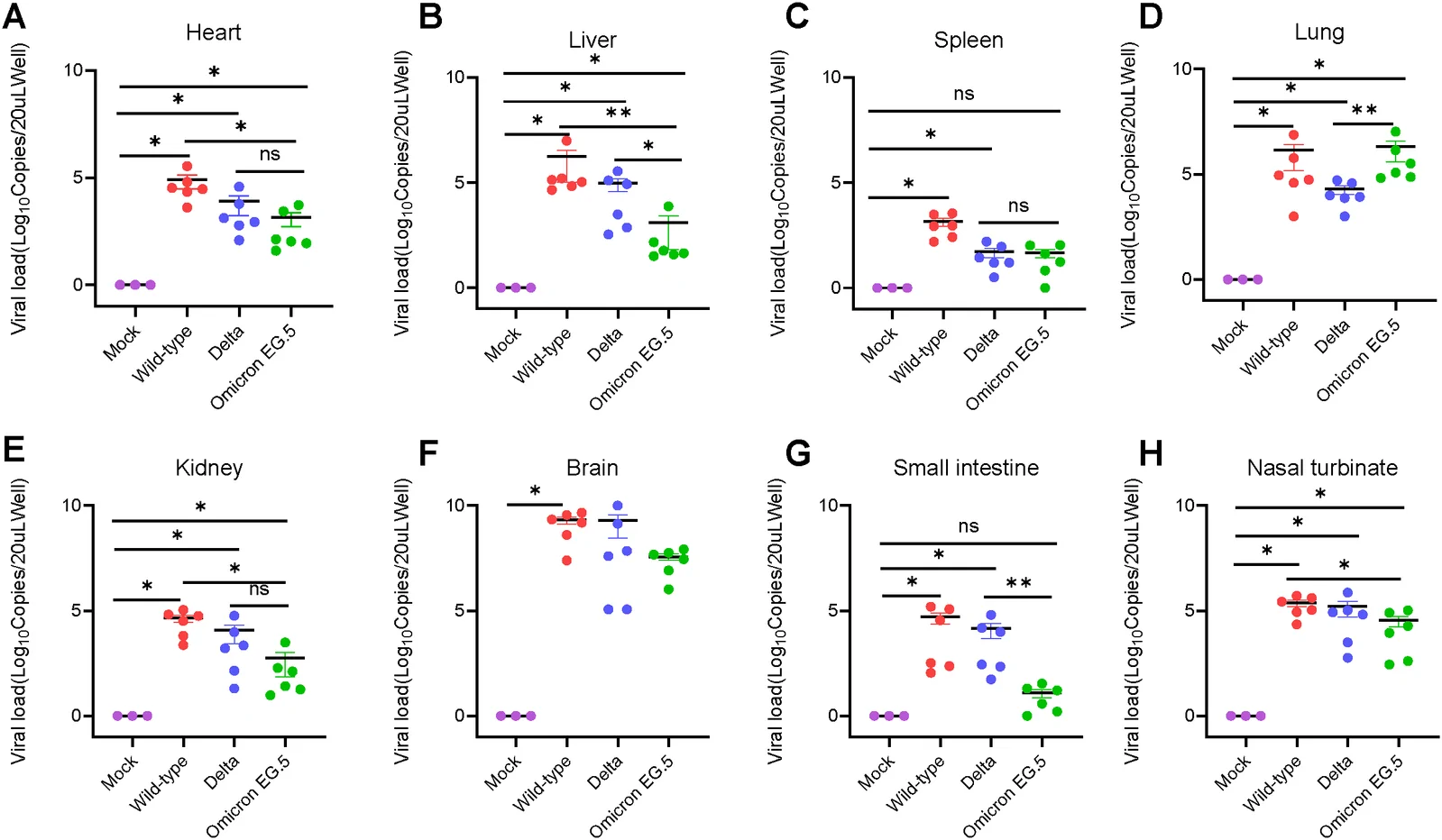
Viral loads in multiple tissues of mice infected with different SARS-CoV-2 variants. Viral loads in heart **(A)**, liver **(B)**, spleen **(C)**, lung **(D)**, kidney **(E)**, brain **(F)**, small intestine **(G)**, and nasal turbinate **(H)** tissues from mice infected with the wild-type strain, Delta variant, Omicron EG.5 variant, or mock-infected control were quantified and expressed as log_10_ copies per 20 μL well. Data are presented as individual data points with mean ± standard error of the mean (SEM). Mock group n=3, Wild-type, Delta, and Omicron EG.5 groups n=6. Asterisks indicate statistically significant differences compared with the mock group: *P < 0.05, **P < 0.01.

To investigate whether the continuous evolution of SARS-CoV-2 leads to a gradual decline in its *in vivo* replication capacity across various organs, we collected tissue samples for droplet digital RT-PCR (dd-RT-PCR) analysis. Quantification of viral RNA loads in eight organs revealed evidence of viral dissemination and replication among the SARS-CoV-2 strains (**Figure 6**). The ancestral wild-type strain consistently achieved the highest viral titers across all examined tissues, indicating robust replication and spread. Notably, it demonstrated particularly robust replication in the brain, where viral loads were significantly higher than those in mock-infected controls (**Figure 6F**). High viral burdens were also detected in the heart, liver, spleen, lung, kidney, intestine, and nasal turbinate. The Delta variant exhibited an intermediate replication phenotype (**Figures 6A–H**). Its overall viral loads were lower than those of the wild-type strain but higher than those of Omicron EG.5. Similar to the ancestral wild-type strain, Delta replicated efficiently in the lungs and nasal turbinates. A significant viral load was also present in the brain tissue compared with mock controls. The Omicron EG.5 variant showed the most attenuated replication profile, with the lowest overall viral RNA copy numbers. However, consistent with the other strains, its replication was detectable and exhibited relative tropism for the lungs, brain, and nasal turbinates, where viral loads were measurably elevated (**Figures 6D, F, H**).

In tissues such as the heart, liver, spleen, kidney, and intestine, all three strains replicated to lower levels than in the core respiratory and neurological tissues. The differences in viral load among the strains in these organs were less pronounced. As expected, no viral RNA was detected in any tissue from mock-infected control mice (**Figure 6**).

## Discussion

The SARS-CoV-2 pandemic has been defined by a dynamic interplay between viral evolution and host adaptation. Our study provides a comprehensive, head-to-head comparative analysis of three pivotal strains - the ancestral Wild-type, the hypervirulent Delta, and the contemporary Omicron EG.5 - within a standardized K18-hACE2 model. The key finding is the establishment of a clear, quantifiable virulence gradient (Wild-type > Delta > Omicron EG.5), validating the attenuated pathogenicity of Omicron EG.5 observed in clinical settings. However, the most salient biological insight from our data lies not merely in this gradient, but in the elucidation of an “evolutionary paradox”: while selective pressures have driven Omicron EG.5 toward reduced cytopathogenicity to enhance transmissibility and immune evasion, this attenuation does not equate to a loss of systemic fitness. Contrary to the notion that reduced severity correlates with restricted replication, we demonstrate that Omicron EG.5 retains a remarkable capacity for multi-organ dissemination, particularly within the central nervous system and gastrointestinal tract. This suggests a strategic decoupling of clinical virulence from tissue tropism, wherein the virus preserves critical reservoirs for persistence even as it moderates disease induction. These findings advance our understanding beyond mere mortality metrics, suggesting that the evolutionary success of Omicron variants relies on maintaining a delicate balance between attenuated pathogenesis and conserved extrapulmonary replication.

The observation that Omicron EG.5 infection led to delayed mortality, attenuated clinical symptoms, reduced viral loads, and less severe histopathological damage aligns with extensive epidemiological data reporting decreased intrinsic severity of Omicron lineages in human populations (12, 14, 17). It is important to note that while we document reduced viral loads and histopathological damage, our current study focuses on viral replication and structural pathology; we did not perform quantitative cytokine profiling or deep immune cell phenotyping. Although our current study focused on viral replication and histopathology, the observed attenuation likely stems from a combination of altered viral entry and differential host immune responses. While our *in vivo* data do not directly interrogate viral entry mechanisms, we speculate that this attenuated phenotype may be partly attributed to previously reported shifts in cellular protease utilization. The ancestral Wild-type and Delta strains predominantly utilize TMPRSS2 for efficient plasma membrane fusion (18, 22, 25), a feature that potentially underlies their high viral loads in the lungs (**Figure 6**)-an organ with abundant TMPRSS2 expression-and their capacity for rapid systemic dissemination. In contrast, the significantly lower viral burden of Omicron EG.5 across examined tissues, particularly in the lungs, is consistent with published reports suggesting that Omicron lineages favor TMPRSS2-independent, endosomal entry pathways (18, 23, 26). Thus, while we observe reduced replication, the extent to which altered protease usage drives this phenotype remains speculative within the context of our current study. Future cell-based studies, such as pseudovirus entry assays, are essential to validate these putative mechanisms.

Despite this attenuation, Omicron EG.5 retained two critical pathogenic features. First, it maintained a pronounced tropism for the intestinal tract, a characteristic shared with the Wild-type and Delta strains, as evidenced by significant viral antigen expression and RNA loads. This conserved gastrointestinal replication underscores a persistent potential for fecal-oral transmission and highlights the gut as a significant viral reservoir (24, 27). Second, and of particular concern, all three strains replicated efficiently in the brain. The high viral RNA loads detected in brain tissue, consistent with other reports (24, 25), confirm the preserved neuroinvasive potential of SARS-CoV-2 across its evolution, a property that may contribute directly to fatal outcomes in this model and merits further investigation regarding its role in human neuropathology. It should be noted that the K18-hACE2 mouse model expresses the human ACE2 receptor under a cytokeratin-18 promoter, which may lead to non-physiological expression patterns and thus potentially lead to an overestimation of neuroinvasion. Therefore, confirmatory studies in other models are warranted to validate our findings regarding brain involvement.

The differential tissue damage observed among the variants likely reflects not only viral replication efficiency but also the host’s inflammatory response. While we documented extensive histopathological lesions, particularly in the lungs and intestines of WT and Delta groups, we acknowledge that the absence of quantitative cytokine profiling and detailed immune cell phenotyping limits our ability to fully dissect the immunopathological mechanisms driving these differences. It is plausible that the reduced tissue damage in EG.5-infected mice results from a decoupling of viral presence from excessive inflammation, a hypothesis supported by reports of altered cytokine induction by Omicron variants (26). Future studies incorporating multiplex cytokine analysis and assessment of immune cell infiltration in target tissues are necessary to delineate whether the attenuated phenotype of EG.5 is primarily driven by intrinsic viral replication defects or a modulated host immune response.

The K18-hACE2 mouse model proved instrumental in revealing this pathogenic spectrum. Its sensitivity, driven by ubiquitous hACE2 expression, allowed for the clear demarcation of virulence differences that might be obscured in less susceptible models. Its utility in modeling severe COVID-19 complications, as demonstrated here and in prior studies of thrombosis (26) and viral co-infections (27), remains unequivocal. However, this very sensitivity is also a limitation of our study. The uniform lethality observed does not reflect the spectrum of disease severity seen in humans and precludes the study of convalescence and long-term sequelae. Furthermore, our analysis focused on an early, acute time point; a longer-term observation might provide additional insights into the resolution of infection, particularly for the attenuated Omicron EG.5 variant.

The differences in clinical severity and tissue damage observed among the variants are likely tightly linked to divergent host immune responses. Ancestral SARS-CoV-2 strains are known to trigger profound cytokine storms and robust macrophage/neutrophil infiltration, contributing to acute respiratory distress and multi-organ failure (28). The attenuated tissue damage and delayed mortality in the Omicron EG.5-infected group, despite the presence of viral RNA in multiple organs, suggest a potentially dampened or delayed pro-inflammatory cytokine response. Rong N et al. found that Omicron BA.1 infection downregulated genes associated with neutrophil pro-inflammatory function (Mmp8, S100a8, S100a9) and attenuated the activation of IL-17 and NF-κB pathways in pro-inflammatory monocytes and pulmonary interstitial macrophages (29). These immunological features are believed to be associated with the attenuated pathogenicity of the Omicron lineage. However, defining the precise immunological landscape requires further investigation.

An important consideration arising from our data is the interpretation of multi-organ viral detection versus systemic infection. While we detected viral RNA and antigen in multiple extrapulmonary tissues across all strains, we caution that these findings represent evidence of viral dissemination and replication rather than definitive proof of independent, productive systemic infection in every sampled organ.​ Several factors may contribute to this distinction. First, RT-ddPCR detects both replicating genomes and residual, non-infectious viral RNA; the latter may persist in tissues following clearance or represent passive seeding during viremia without subsequent rounds of replication. Second, while immunohistochemical detection of nucleocapsid protein confirms viral protein translation, it does not unequivocally distinguish between abortive infection and productive viral cycles. Third, the widespread expression of hACE2 in the K18 transgenic model may facilitate broader viral distribution compared to natural human infections. Therefore, the observed multi-organ involvement should be interpreted as widespread viral tropism and dissemination, potentially including both productive and non-productive encounters, rather than classical hematogenous systemic infection. Future studies utilizing subgenomic RNA analysis or *in situ* hybridization for negative-sense viral RNA will be essential to definitively differentiate between these possibilities and to map the precise cellular dynamics of SARS-CoV-2 dissemination.

In conclusion, our data provide experimental validation of the attenuated pathogenicity of the Omicron EG.5 variant within a controlled, comparative framework. Notably, we observed persistent viral detection in the brain and intestinal tissues, suggesting that certain tissue tropisms - particularly involving the central nervous system and gut - may have been retained throughout viral evolution. However, caution is warranted when interpreting these findings, as the K18-hACE2 model recognized limitations for assessing neurotropism due to the non-physiological expression of the transgene. These findings reinforce the necessity of ongoing surveillance that tracks not only transmissibility and immune evasion, but also the fundamental pathogenic potential of emerging variants. Understanding the mechanistic basis for this continued attenuation, including the complex interplay between viral replication and host immune responses, will be crucial for guiding long-term public health preparedness.

## Limitations of the study

It is important to note the limitations of our work. First, the K18-hACE2 mouse model, while highly valuable for pathogenesis studies, exhibits extreme susceptibility leading to uniform lethality, which does not fully recapitulate the wide spectrum of disease severity observed in humans. Second, our analysis was focused on the acute phase of infection (3 dpi and survival endpoint); longer-term observations could provide insights into recovery or prolonged pathology. Finally, the use of a single, high challenge dose may not reflect transmission-level exposures. Future studies employing models with regulated hACE2 expression or investigating lower, more physiological inoculum could further elucidate variant-specific pathogenesis.

## Data Availability

The original contributions presented in the study are included in the article/supplementary material. Further inquiries can be directed to the corresponding author.
